# In Silico Characterization of RNASEH2A Pathogenic Variants and Identification of Novel Splice Site Donor Variant c.549+1G>T in Indian Population

**DOI:** 10.7759/cureus.40366

**Published:** 2023-06-13

**Authors:** Vykuntaraju K Nanjundagowda, Swabhiman Paikaraya, Varunvenkat M Srinivasan, Anshika Srivastava

**Affiliations:** 1 Pediatric Neurology, Indira Gandhi Institute of Child Health, Bengaluru, IND; 2 Division of Medicinal and Process Chemistry, Council of Scientific and Industrial research-Central Drug Research Institute (CSIR-CDRI), Lucknow, IND; 3 Medical Genetics, Sanjay Gandhi Postgraduate Institute of Medical Sciences, Lucknow, IND

**Keywords:** protein modeling, india, splicing variant, rnaseh2a gene, aicardi-goutieres syndrome

## Abstract

Background

Aicardi-Goutieres syndrome (AGS) is a genetic disorder that has variable manifestations including neurological, immunological, and sometimes other system involvement in various combinations. Considering the high genetic and clinical diversity of AGS and the importance of RNASEH2 complex in the biological system, it is important to take a systematic approach to delineate the genetic diagnosis and impact of missense mutations.

Methods

Clinical targeted gene sequencing followed by Sanger validation was performed in an individual with the clinical features of AGS. Protein modeling studies of all the reported *RNASEH2A* missense variants till date were performed using freely available web servers BioGrid, ShinyGO. Protein structures were visualized using Pymol.

Results and discussion

We identified a novel homozygous splice site donor variant c.549+1G>T in *RNASEH2A. *Furthermore protein-interactome studies identifiedpotential genetic interactors that include *RNASEH2A, RNASEH2B, TYMS, RNASEH2C, RPA1, ORC3, ORC2, CDC6, PCNA, LIG1, PRIM1, RFC2, DUT, GINS1, MCM7, FEN1, MCM4, GINS2, CDK4, *and* MCM5*. Identified genes were mapped to specific pathways using SHINY GO. DNA replication and cell cycle, centrosome cycle, post-replication repair, nucleic acid and metabolic process, cellular response to stress, DNA metabolic process, nucleic acid phosphodiester bond hydrolysis, RNA phosphodiester bond hydrolysis, and DNA biosynthetic process were identified as the linked pathways with the prioritized genes.

Conclusion

In conclusion, a sophisticated genotype and phenotype correlation followed by linking the genes to the key biological pathways opens new avenues to understand disease pathology and plan for therapeutic interventions.

## Introduction

Aicardi-Goutières syndrome (AGS) is a neuroinflammatory autoimmune disease characterized by severe brain dysfunction (encephalopathy), skin lesions, and other health issues linked to aberrant immune system activation [1,2]. AGS occurs in about 1 in 105,000 to 167,000 newborns in the United States. Although actual prevalence of AGS is unknown in India however, worldwide estimate suggests that there are approximately 4,000 affected individuals. Pathogenic variants in genes encoding several nucleotide-processing proteins as TREX1 (OMIM 225750), RNASEH2A (OMIM 606034), RNASEH2B (OMIM 610326), RNASEH2C (OMIM 610330), SAMHD1 (OMIM 606754), ADAR (OMIM 146920), IFIH1 (OMIM 606951), LSM11 (OMIM617910), RNU7-1 (OMIM 617876) have been found in affected individuals of all ethnic origins [3]. The disease is mostly inherited in an autosomal recessive manner except for AGS1 and AGS6 caused due to mutations in TREX1, ADAR that can have both recessive and dominant inheritance, and AGS7 caused due to mutations in IFIH1 inherited in an autosomal dominant manner [4-6].

The clinical features in AGS include global developmental delay, regression of milestones, microcephaly, seizures, dystonia, and spasticity to variable degree. The most common systemic manifestations include hepatosplenomegaly, chilblain lesions, and sterile pyrexia [7-10]. The characteristic neuroimaging findings on MRI include white matter abnormalities like demyelination and delayed myelination. The grey matter abnormalities include cerebral atrophy. Intracranial calcifications are common [9,11]. The usual clinical course is described as encephalopathy of sub-acute type followed by more regression of milestones and microcephaly which may last for several months. Finally, the patients develop spastic tetraplegia in what is termed the stabilization phase [11].

Autosomal recessive mutations in RNASEH2A lead to Aicardi-Goutieres syndrome type-4 (OMIM#610333). RNASEH2A, together with RNASEH2B and RNASEH2C, constitutes the holoenzyme ribonuclease (RNase) H2 [12,13]. RNASEH2B and RNASEH2C are used as the scaffold for RNASEH2A, which serves as the catalytic subunit of the RNase H2 complex [14]. As a ribonuclease, this complex aids in the breakdown of RNA-DNA hybrid, created during DNA replication, specifically targeting the breakdown of RNA molecules that are chemically related to DNA. Additionally, RNase H2 complex is involved in DNA replication [15], error repair [16], and other cellular processes, including helping to prevent inappropriate immune system activation [3]. Disturbance in RNase H activity affects microRNA turnover, resulting in severe clinical consequences in the brain that characterize the clinical feature of AGS [17]. Knockout mouse model Rnaseh2aG37S, a hypomorphic mutation, producing an enzyme with partial loss of both RNA/DNA hydrolysis and ribonucleotide excision repair (RER), causes perinatal lethality whereas homozygous G37S embryos are smaller from an early embryogenesis period of E10.5, present at expected Mendelian ratio [18]. Moreover, Rnaseh2aRED, which is ribonucleotide excision defective but active for RNA/DNA hybrid processing when crossed with Rnaseh2aG37S, became embryonic lethal at E12.5 in Rnaseh2aG37S/RED mice suggesting the dominant role of Rnaseh2aRED enzyme [19]. However, our understanding of the precise impact of missense variants of RNASEH2A and AGS disease severity is far from complete.

Various methods can be used to predict the effect of missense mutations on protein stability, based on either structural [20-22] or sequence data [23,24]. However, structural approaches often assume that proteins are static and do not consider the effects of a mutation within their conformational landscape. Despite this limitation, the information obtained from both strategies is often complementary. Here, we identify a novel homozygous splice site donor variant in c.549+1G>T in an individual who presents with core features of AGS4, including regression of developmental milestones in all domains following trauma. In order to understand the impact of missense mutations on protein stability and dynamics we used a sophisticated pipeline of enhanced servers that combines normal mode analysis with graph-based representation of protein structure reported in RNASEH2A till date. This particular approach will accurately and quickly predict the effects of single and multiple-point mutations on protein stability and dynamics. To the best of my knowledge, this is the first report that identified a novel splice site donor in RNASEH2A and used the protein modeling tools to study the impact of missense mutations published till date in RNASEH2A.

## Materials and methods

Ethics statement and clinical sample collection

The present study is a part of an ongoing research project (IEC Code: 2020-163-EMP-EXP-21) that started in the year 2020 to recruit individuals with inherited neurodevelopmental disorders. A total of 100 consanguineous families from Southern India have been recruited to date. The parents provided written informed consent for the clinical exome assay. Blood samples were collected from all three members of the family (father, mother, proband) and DNA was isolated from peripheral blood by Qiagen DNA extraction kit according to the manufacturer’s instructions (Qiagen, Valencia, CA, USA). The quality of DNA was assessed by agarose gel electrophoresis followed by Nanodrop quantification (Nanodrop 2000, Thermo Scientific, Waltham, MA, USA).

Clinical features of proband

**Figure 1:**
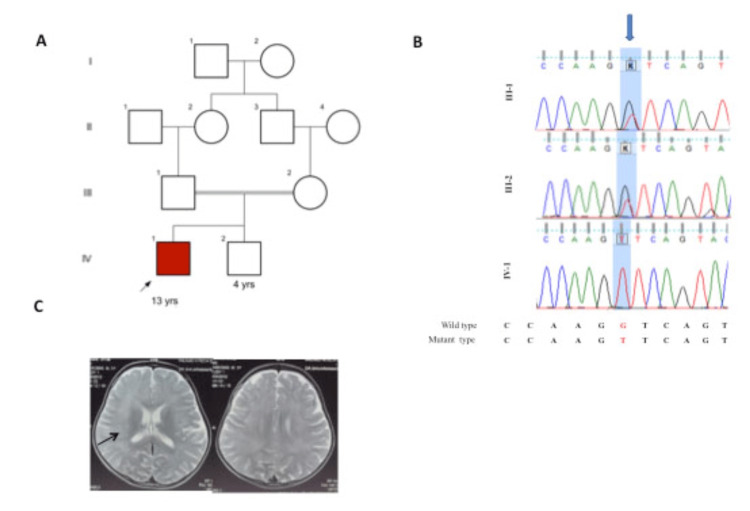
Brain imaging and RNASEH2A variant in proband (1A) showing pedigree, (1B) showing Sanger sequencing in proband and parents, (1C) MRI Brain plain showing mild T2 hyperintensities in periventricular white matter (Arrows).

The proband is a 13-year-old boy born to a 3rd-degree consanguineous married couple (Figure 1A) in South India with complaints of gradual regression of attained milestones post minor trauma at three years of age. Antenatal history was unremarkable. He is born to full-term normal vaginal delivery with a birth weight of 2.7 kg; cried immediately after birth. Developmentally, the child had attained all milestones as per age until 3 years, that is, he could ride a tricycle, could draw a circle, knew his full name, and could identify his gender. At the age of three years, post an accidental fall with minor trauma followed by a brief loss of consciousness for 15 minutes, with no history of seizures, vomiting, and irritability. Following 15 days post-trauma, he had gradual regression of milestones in all domains. Initially, the child was not able to walk, and over a period of two days, lost sitting and finally neck control. His speech became slurred, and he could not speak after two days. Difficulty in feeding was seen in the form of slow feeding. Clinical examination at this point of time revealed a head circumference of 49 cm (0.35 WHO Z score), a weight of 11 kg (-1.85 WHO Z score), and a length of 90 cm (-1.33 WHO Z score). No neurocutaneous markers were evident. Higher mental function revealed a conscious patient with no speech. The cranial nerves system was normal including a normal pupillary response. The motor system showed normal bulk, spasticity of all limbs, and power of 3/5 in all limbs with brisk deep tendon reflexes and extensor plantar. Skull, spine, and other system examinations were normal. No meningeal signs. Fundus examination and hearing assessment was normal.

Investigations including complete hemogram, liver function, renal function, serum ammonia, serum lactate, serum electrolytes, and arterial blood gas were all normal. Cerebrospinal fluid (CSF) examination showed five lymphocytes with normal sugar and protein with sterile cultures for bacterial, and fungal microbes. CSF polymerase chain reaction for common viral infectious agents was also negative. Brain MRI showed symmetric T2 hyperintensities in bilateral periventricular white matter (Figure 1B). A possibility of metachromatic leukodystrophy was kept and further, nerve biopsy and arylsulfatase A levels were performed but they were normal. He was admitted for a period of 15 days for the same, treated and managed symptomatically. The child was managed as genetic leukodystrophy; however, no genetic testing was done due to cost constraints. At the time of discharge, the child had no neck control, had severe spasticity with no speech. A trial of steroids was to be tried but parents refused any further treatment and subsequently were lost to follow up. The child was advised physiotherapy and speech therapy; the child gained motor milestones at five years of age. Initially, he started sitting at five years and five months, standing by six years and five months, and started walking with support by eight years and now uses a walking stick for support. He started speaking a few words by six years of age and now can speak sentences and tell stories with mild dysarthria. The family next visited at nine years. The child needed support in walking with a stick, had dysarthria and could thrive for his daily activities with some support, and was attending regular school with good performance. Based on the clinical features the only proband sample was sent for clinical exome array.

Clinical targeted gene sequencing

DNA extracted from blood was used to perform targeted gene capture using a custom capture kit. The libraries were sequenced to mean >80-100X coverage on Illumina sequencing platform. MedGenome Labs Ltd. (Bangalore, India) performed the clinical targeted gene sequencing. Selective capture and sequencing of the protein-coding regions of the genome/genes is performed. Mutations identified in the exonic regions are generally actionable compared to variations that occur in non-coding regions. Targeted sequencing represents a cost-effective approach to detect variants present in multiple/large genes in an individual.

Variant calling and filtering

The FastQ sequences obtained were aligned to the human reference genome (GRCh37/hg19) using the BWA program and analyzed using Picard and GATK version 3.6 to identify variants relevant to the clinical indication. GATK best practices framework was followed for the identification of variants in the sample. Gene annotation of the variants is performed using the VEP program against the Ensembl release 87 human gene model. Clinically relevant mutations were annotated using published variants in literature and a set of diseases databases - ClinVar, OMIM, GWAS, HGMD and Swiss Var. Common variants are filtered based on allele frequency in 1000 Genome Phase 3, ExAC, EVS, dbSNP147, and our internal Indian population database. Non-synonymous variants effect is calculated using multiple algorithms such as PolyPhen-2, SIFT, Mutation Taster2, Mutation Assessor, and LRT. Only non-synonymous and splice site variants found in the clinical exome panel consisting of 8332 genes were used for clinical interpretation. Silent variations that do not result in any change in amino acid in the coding region are not reported.

Sequence-based analysis and protein modeling

Multiple sequence alignment of RNASEH2A across different species was performed using MAFFT tool, a web-based tool made to simplify the creation of sequence logos. With the support of multiple sequence alignments (MSA), we created a phylogenetic tree. We compiled a set of gene or protein sequences from various organisms. Using MAFFT sequence alignment tool, sequences were aligned. The CLUSTAL format alignment by MAFFT results was used in the alignment process to make sure that corresponding places in the sequences are matched correctly, accounting for gaps and mismatches. Then we downloaded the tree file in the Newick format after receiving the result. In order to obtain the phylogenetic tree, which illustrates the evolutionary relationships among the organisms, we uploaded the tree file to the ITOL web server.

To create a weblogo representation, firstly, we gathered a set of RNASEH2A sequences from different organisms. Then, using the sequence alignment tool MAFFT, sequences were aligned. The alignment process ensures that corresponding positions in the sequences are aligned properly, taking gaps and mismatches into account, which we get from CLUSTAL format alignment by MAFFT. Then we put the CLUSTAL format alignment on the WebLogo web server and showed that RNASEH2A gene is conserved in the diverse kingdom of life. For RNASEH2A protein modeling we used the crystal structure, PDB ID - 3P56. We have highlighted the mutation on the crystal structure of the trimer of RNASEH2A, RNASEH2B, and RNASEH2C that we took from the protein data bank (PDB). Pymol tool was used for the 3D visualization of protein. To examine the impact of single and multiple point mutations on protein stability and dynamics, DYNAMUT2, a web server that combines Normal Mode Analysis (NMA) methods to capture protein motion and graph-based signatures to represent the wild-type environment was used.

Protein-protein interaction network and pathway

To predict the functional interactions of RNASEH2A, we used (STRING) database (https://string-db.org). The protein-protein interaction (PPI) networks were built using active interaction sources such as text mining, experiments, databases, co-expression, and species limited to "Homo sapiens". In networks, the edges stand in for interactions while the nodes represent the proteins.

Functional enrichment analysis

For the functional enrichment analysis, we used ShinyGO. It is a web-based tool that allows users to explore and analyze the functional enrichment of gene sets or proteins in biological pathways and gene ontology terms. While ShinyGO primarily focuses on gene ontology analysis, it can also provide insights into the relationship between protein-protein interactions (PPIs) and biological pathways.

By using ShinyGO's functional enrichment analysis, we can identify biological pathways that are significantly enriched with the input proteins based on available gene ontology annotations. This can help to understand the functional context and potential roles of the proteins in specific pathways. However, it's important to note that ShinyGO primarily focuses on gene ontology analysis and may not directly provide information on specific PPIs within those pathways.

## Results

Variant prioritization

Through clinical targeted gene sequencing, we identified a homozygous novel 5’ splice site variant at chr19-12921023: c.549+1G>Tin intron 5 of RNASEH2A with depth 64x. The splice variant affects the invariant GT donor splice site of exon 5. In-silico prediction of the variant confirmed damaging by MutationTaster2. varSEAK online tool (https://varseak.bio/) showed that genetic variant c.549+1G>T leads to loss of function of the authentic donor site and use of cryptic site 66 nucleotide upstream of 5’ splice site. The parents were heterozygous for both the variants identified in proband, as confirmed by Sanger sequencing and consistent with consanguinity (Figure 1C). Sanger sequencing was performed at ABI 3500 Genetic Analyzer (Applied Biosystems Inc., Waltham, MA, USA) with 50 cm long and 50 mm diameter capillary containing Performance-Optimized Polymer-7 (POP-7, Applied Biosystems). The samples were injected into the capillary at the voltage of 15 kV for 40 minutes at 60ºC. Amplicon size of separated fragments was calculated against GS500-LIZ size standard using the GeneMapper. According to the variant classification guidelines of ACMG, genetic variant c.549+1G>T is categorized as “likely pathogenic” (PVS1: Pathogenic strong; Null variant in a gene where loss of function is a known mechanism of disease, PM2: Pathogenic Moderate; Extremely low frequency in gnomAD population databases).

Sequence-based analysis

**Figure 2:**
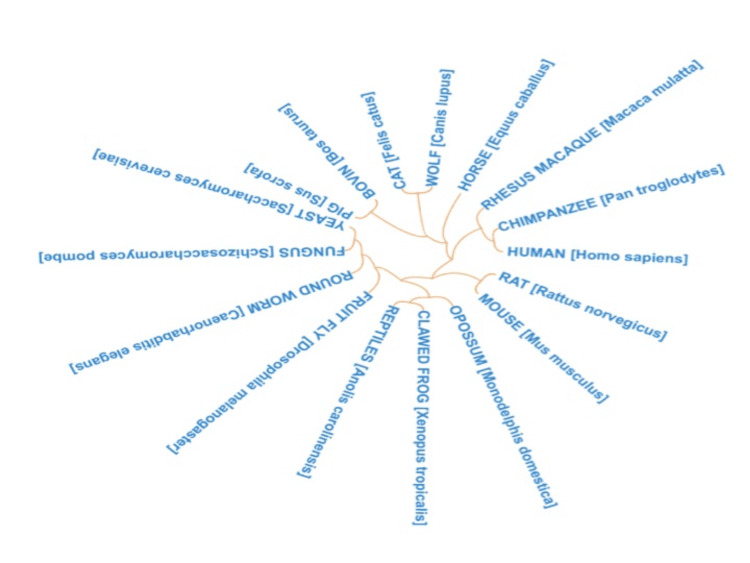
Phylogenetic tree of RNASEH2A conservation across mammalian species Phylogenetic tree showed the high conservation of RNASEH2A across mammalian species.

**Figure 3:**
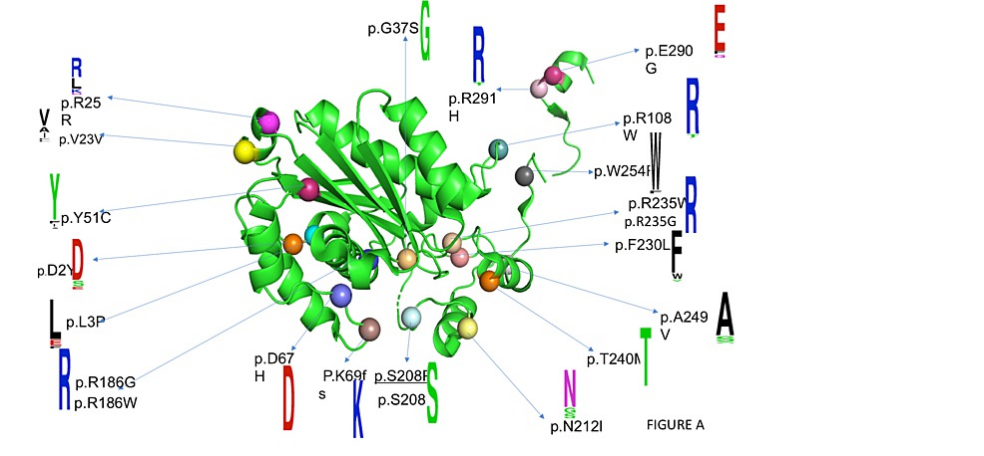
Web logos of RNASEH2A variants were mapped to protein structure. The amino acid residues in the wild-type RNASEH2A are highly conserved.

Sequences of RNASEH2A of mammalian species were fetched from HGNC web database. Phylogenetic tree showed the high conservation of RNASEH2A across mammalian species (Figure 2). To know the sequence level conservation of pathogenic variant, we created a web logo post-homology modeling. The web logos of RNASEH2A variants were mapped to protein structure as shown in Figure 3. The amino acid residues in the wild-type RNASEH2A were highly conserved and therefore mutation in these conserved positions lead to the clinical phenotype of AGS (Figure 3).

Protein structural analysis

**Figure 4:**
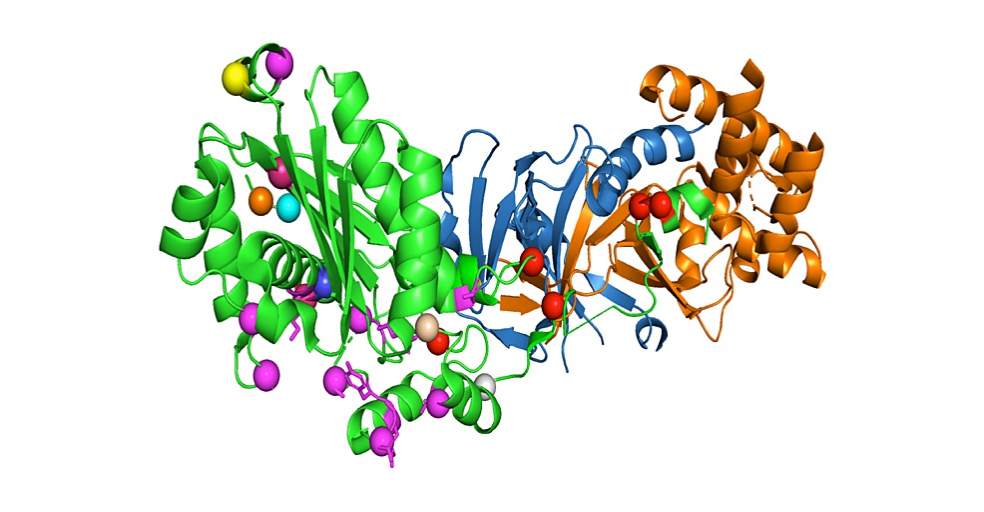
3D structure of the RNaseH2 protein The spheres denote the mutation points. The mutations are mostly found in the A subunits, i.e., D2Y, L3P, V23V, R183G, R235W, E290G, etc. Some of the mutations occur at the interface of subunit A and 3, which is shown in the red colour sphere and some of the mutations occur in the active pocket of the protein, which may affect the functionality of the protein, which is shown in the magenta colour sphere. Splice mutation sites are colored and marked as stars.

The three subunits of human RNaseH2 protein complex are arranged in a line, with the subunit B and A on either side of the central subunit C. RNaseH2 is crystallized as dimer of trimers forms a dimer with a core 14-stranded triple-barrel fold, the B and C subunits combine. The catalytic A subunit interacts almost exclusively with subunit C. A α-helix (residues 98-108) and three loops (residues 196-199, 227-232, and 250-256) of the A subunit make mostly hydrophobic contacts with four regions of the C subunit (residues 34-36, 47-51, 62- _67, and 134-135). The extreme C-terminal fragment of subunit A (residues 284-299) also forms extensive contacts with subunits B and C, and residues 292-296 add a short β _strand to the central β sheet of the triple barrel of subunit B and C. In the 3D structure of the RNaseH2 protein, the spheres denote the mutation points. The mutations are mostly found in the A subunits, i.e., D2Y, L3P, V23V, R183G, R235W, E290G, etc. Some of the mutations occur at the interface of subunit A and 3, which is shown in the red colour sphere in Figure 4, and some of the mutations occur in the active pocket of the protein, which may affect the functionality of the protein, which is shown in the magenta colour sphere in Figure 4. Splice mutation sites are colored and marked as stars.

The mutations that occur at the subunit A and C interface, i.e., R108W, F230L, W254R, E290G, and R291H, impair RNAH2 complex stability and therefore the function. The G37S mutation, located in the active pocket and is part of the GRG motif, impairs substrate cleavage. Another mutation found in the active pocket is T240M. Thr240 plays a key role in the binding of the non-cleaved strand, and its substitution with alanine can significantly lower the enzyme activity. We calculated free energy change on account of mutation using DUET and mCSM webservers (Supplementary Table 1) and the results indicate that most of the mutations are destabilizing the structure and therefore further support the observed pathogenicity in the patient.

**Figure 5:**
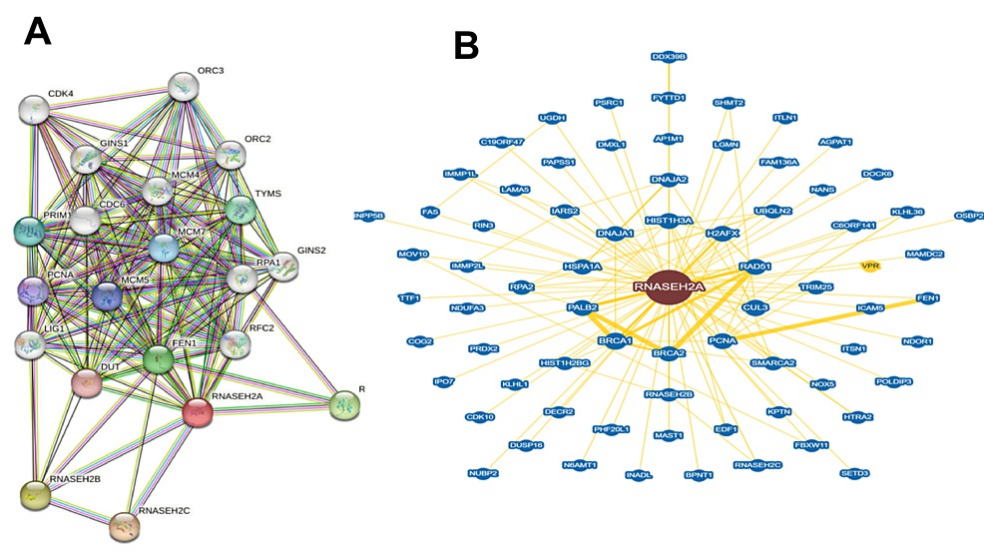
Protein-protein interactors STRING and BioGrid databases identified that RNASEH2A interacts with RNASEH2B, TYMS, RNASEH2C, RPA1, ORC3, ORC2, CDC6, PCNA, LIG1, PRIM1, RFC2, DUT, GINS1, MCM7, FEN1, MCM4, GINS2, CDK4, and MCM5.

**Figure 6:**
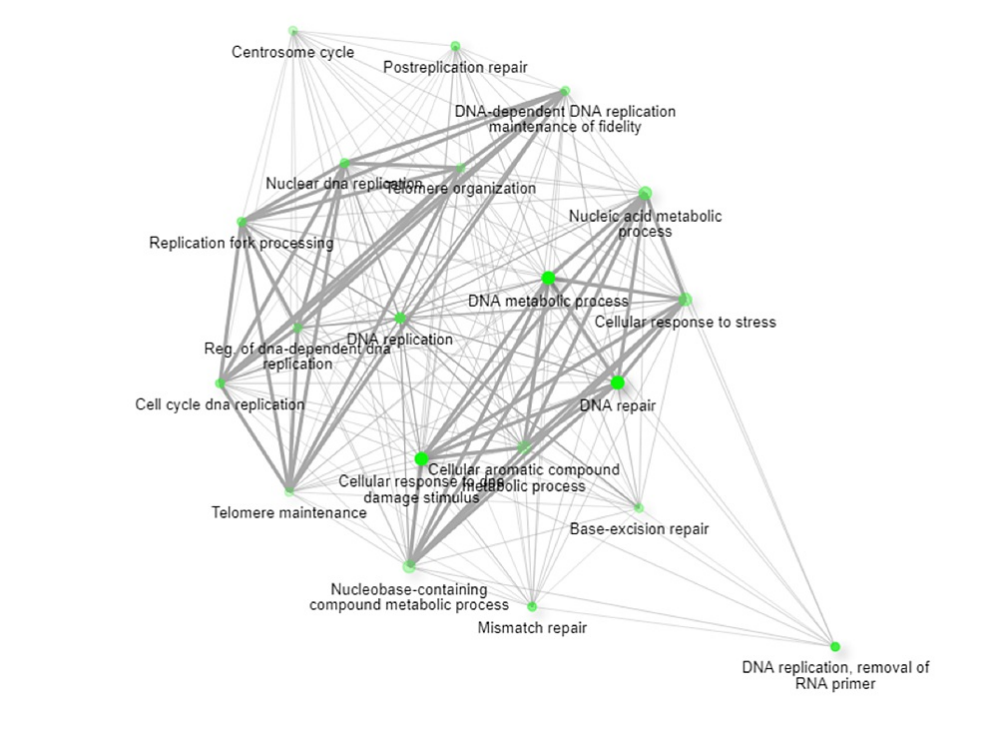
ShinyGO, identified potential pathways that could be misregulated in AGS4 Using ShinyGO, we identified potential pathways that could be misregulated in AGS4. They are DNA replication and cell cycle, centrosome cycle, post-replication repair, nucleic acid and metabolic process, cellular response to stress, DNA metabolic process, nucleic acid phosphodiester bond hydrolysis, RNA phosphodiester bond hydrolysis, and DNA biosynthetic.

To learn about the potential protein-protein interactors, we search STRING and BioGrid databases and the protein-protein interaction networks are presented in Figure 5. The results indicate that RNASEH2A interacts with RNASEH2B, TYMS, RNASEH2C, RPA1, ORC3, ORC2, CDC6, PCNA, LIG1, PRIM1, RFC2, DUT, GINS1, MCM7, FEN1, MCM4, GINS2, CDK4, and MCM5. Further, these genes were mapped to specific pathways using SHINYGO, which can lead to the identification of key biological pathways that can be affected in patients (Figure 5). Data analysis further demonstrates that DNA replication and cell cycle, centrosome cycle, post-replication repair, nucleic acid and metabolic process, cellular response to stress, DNA metabolic process, nucleic acid phosphodiester bond hydrolysis, RNA phosphodiester bond hydrolysis, and DNA biosynthetic process could be potentially affected in patient which may lead to pathogenesis (Figure 6).

## Discussion

This study implicates a novel splice site donor variant in RNASEH2A in the genetic etiology of AGS-4. We identified the interactome through STRING and BioGrid webserver. Results indicated the potential interacting genes that include RNASEH2A, RNASEH2B, TYMS, RNASEH2C, RPA1, ORC3, ORC2, CDC6, PCNA, LIG1, PRIM1, RFC2, DUT, GINS1, MCM7, FEN1, MCM4, GINS2, CDK4, and MCM5. Further, these genes were mapped to specific pathways using ShinyGO. DNA replication and cell cycle, centrosome cycle, post-replication repair, nucleic acid and metabolic process, cellular response to stress, DNA metabolic process, nucleic acid phosphodiester bond hydrolysis, RNA phosphodiester bond hydrolysis, and DNA biosynthetic process were identified as the linked pathways with the prioritized genes. This pipeline could successfully map the RNASH2A gene with key biological pathway.

RNASEH2 is a key nuclear enzyme primarily responsible for degrading the RNA strand in RNA/DNA hybrids and breaking down the 5' phosphodiester bond of ribonucleotides embedded in DNA duplexes [20,21]. The interaction between the PIP-box of RNASEH2B and PCNA can enhance these enzymatic activities. Eukaryotic cells often experience the frequent misincorporation of single ribonucleotides into genomic DNA-by-DNA polymerases [22]. If left uncorrected, this can potentially lead to genome instability [23]. RNase H2 also possesses the ability to cleave R-loops, which are stable RNA/DNA hybrids formed during transcription [24].

Within the RNase H2 enzyme complex, the active site residues are found in the RNASEH2A subunit, while the B and C subunits are believed to serve as a platform for complex assembly and regulate its interactions with other proteins. Structural studies of mouse [25] and human [26,27] RNase H2 complexes have revealed that the RNASEH2B and C subunits form a tightly intertwined dimer. Additionally, the C-terminal extension of the catalytic RNASEH2A subunit interacts with this dimer and is crucial for the formation of an active RNase H2 trimer.

In silico predictions suggested that genetic variant c.549+1G>T leads to loss of function of the authentic donor site and use of cryptic site 66 nucleotide upstream of 5’ splice site. Proband in this study presented with gradual regression of milestones in all domains following trauma and had spontaneous recovery over many years with residual deficits in the form of spasticity and dysarthria with normal intellect. AGS unusually presents before or during the first year and has the following phases: encephalitic phase followed by plateau phase. Unusually post the acute insult and during the plateau phase, there are residual symptoms and signs of intellectual impairment of varying degree with spasticity and dystonia. Some patients also may have chilblains and autoimmunity due to abnormal activation. The clinical features, severity and outcomes can be variable even within a family. A study of 374 patients through review with molecularly confirmed AGS with mutations in the seven genes showed that mortality was noted in 67 cases (19.3%), 68 cases (19% survived beyond 15 years) and eight beyond age 30 years [28]. Progression may be variable except in autosomal dominant ADAR p. G1007R spastic paraparesis in some patients, which is progressive. Crow et al. in their study on the characterization of human phenotypes with genes causative of AGS identified 14 families (5%) with mutations in RNASEH2A gene. The most common presentation was between 0-12 months. All these children either had prior abnormal development or were uncertain with none being normal [3]. In another study, four patients were identified with one heterozygous missense variant and the other variant being a synonymous variant with splicing effects either c.69G>A or c.75C>T. The missense heterozygous variants were Patient-1, c.704G>A: p.Arg235Gln, Patient-3, c.556C>T:p.Arg186Trp and Patient-4, c.635A>T: p.Asn212Ile. Functional analysis of these genetic variants showed that both synonymous variants lead to leaky splicing thus allowing the formation of small products [29,30].

Our informatics-based analysis indicates that RNASH2A enzyme is highly functionally conserved across mammalian species. Our sequence level analysis indicated that the sequences that got mutated were highly conserved and mutation can therefore impact on its structure and function. The missense mutations can impact on the stability and activity of RNASEH2A protein, depending on the specific mutation and its location within the protein structure. Many missense mutations in RNASEH2A have been identified in AGS patients, and some of these mutations have been studied to understand their effects on protein stability and dynamics. Further, we identified the potential protein-protein interactors and key biological pathways associated with the interactome. These results will provide a better understanding of genotype to phenotype correlation and foundation for further investigation of molecular basis of key biological pathways, which can be impacted on account of missense variants in patients.

## Conclusions

In conclusion, genotype and phenotype correlation performed by us and others showed the impact of pathogenic mutation on the structure and function of RNASEH2A. Furthermore, we also decoded the molecular mechanism through the identification of interactome and potential biochemical pathways/networks. Therefore, our study provides a platform for biological network-based repurposing in patient cell lines to identify potential drugs to manage the clinical features of AGS. Network-based drug repurposing has the potential to identify new therapeutic applications for existing drugs by leveraging the knowledge of molecular interactions and networks. Repurposed drugs have already been tested for safety and toxicity and they can even bypass some preclinical and early-phase clinical trials. Moreover, acceleration of therapeutic development increases treatment options, and improved outcomes for patients with RNASEH2A-mediated genetic disorders.

## Appendices

**Table 1:** Calculation of energy across reported RNASEH2A variants Predicted Stability Change (ΔΔG) = -ve, i.e., Destabilizing Predicted Stability Change (ΔΔG) = +ve, i.e., Stabilizing

Genetic Variants	DYNAMUT2	MCSM	Post Translational Modification (PTM)
	ΔΔG[Kcal/MOL]	ΔΔG[Kcal/MOL]	
p.Asp2Tyr	0.39	0.238	
p.Leu3Pro	-0.99	-1.283	
p.Val23Val			
p.Arg25Arg			
p.Gly37Ser	-1.22	-1.363	
p.Tyr51Cys	-1.7	-1.778	
p.Asp67His	-0.98	-0.752	
p.Lys69fs			+
p.Arg108Trp	0.17	-0.103	
p.Arg186Trp	-1.38	-1.209	
p.Arg186Gln	-1.82	-1.953	
p.Ser208Pro	-0.42	-0.435	
p.Ser208Leu	-0.41	-0.328	
p.Asn212Ile	0.44	0.135	
p.Phe230Leu	-1.37	-1.107	
